# Pathology-related changes in cardiac energy metabolites, inflammatory response and reperfusion injury following cardioplegic arrest in patients undergoing open-heart surgery

**DOI:** 10.3389/fcvm.2022.911557

**Published:** 2022-07-22

**Authors:** Katie L. Skeffington, Marco Moscarelli, Safa Abdul-Ghani, Francesca Fiorentino, Costanza Emanueli, Barnaby C. Reeves, Prakash P. Punjabi, Gianni D. Angelini, M-Saadeh Suleiman

**Affiliations:** ^1^Bristol Heart Institute and Bristol Medical School, University of Bristol, Bristol, United Kingdom; ^2^National Heart and Lung Institute, Imperial College, London, United Kingdom; ^3^GVM Care & Research, Anthea Hospital, Bari, Italy; ^4^Department of Physiology, Faculty of Medicine, Al-Quds University, Jerusalem, Palestine; ^5^Nightingale-Saunders Clinical Trials and Epidemiology Unit (King's Clinical Trials Unit), King's College London, London, United Kingdom

**Keywords:** cardioplegia, metabolites, coronary artery bypass graft (CABG), aortic valve replacement (AVR), ischaemic reperfusion injury

## Abstract

**Introduction:**

Changes in cardiac metabolites in adult patients undergoing open-heart surgery using ischemic cardioplegic arrest have largely been reported for non-ventricular tissue or diseased left ventricular tissue, with few studies attempting to assess such changes in both ventricular chambers. It is also unknown whether such changes are altered in different pathologies or linked to the degree of reperfusion injury and inflammatory response. The aim of the present work was to address these issues by monitoring myocardial metabolites in both ventricles and to establish whether these changes are linked to reperfusion injury and inflammatory/stress response in patients undergoing surgery using cold blood cardioplegia for either coronary artery bypass graft (CABG, *n* = 25) or aortic valve replacement (AVR, *n* = 16).

**Methods:**

Ventricular biopsies from both left (LV) and right (RV) ventricles were collected before ischemic cardioplegic arrest and 20 min after reperfusion. The biopsies were processed for measuring selected metabolites (adenine nucleotides, purines, and amino acids) using HPLC. Blood markers of cardiac injury (Troponin I, cTnI), inflammation (IL- 6, IL-8, Il-10, and TNFα, measured using Multiplex) and oxidative stress (Myeloperoxidase, MPO) were measured pre- and up to 72 hours post-operatively.

**Results:**

The CABG group had a significantly shorter ischemic cardioplegic arrest time (38.6 ± 2.3 min) compared to AVR group (63.0 ± 4.9 min, *p* = 2 x 10^−6^). Cardiac injury (cTnI release) was similar for both CABG and AVR groups. The inflammatory markers IL-6 and Il-8 were significantly higher in CABG patients compared to AVR patients. Metabolic markers of cardiac ischemic stress were relatively and significantly more altered in the LV of CABG patients. Comparing diabetic and non-diabetic CABG patients shows that only the RV of diabetic patients sustained major ischemic stress during reperfusion and that diabetic patients had a significantly higher inflammatory response.

**Discussion:**

CABG patients sustain relatively more ischemic stress, systemic inflammatory response and similar injury and oxidative stress compared to AVR patients despite having significantly shorter cross-clamp time. The higher inflammatory response in CABG patients appears to be at least partly driven by a higher incidence of diabetes amongst CABG patients. In addition to pathology, the use of cold blood cardioplegic arrest may underlie these differences.

## Introduction

Cardiac surgery using cardiopulmonary bypass and cardioplegic arrest renders the heart globally ischemic and susceptible to the damaging effects of reperfusion injury (1, 2). The resulting myocardial injury is associated with postoperative morbidity and, in the long term, can lead to heart failure. Therefore, the search continues for an optimal cardioprotective cardioplegic solution (3–7). Understandably, most of the studies that have been conducted to assess cardioprotective interventions were done during coronary artery bypass graft (CABG) surgery and were uncritically extended to other pathologies (8, 9). However, it is known that different cardiac disease etiologies/modeling respond differently to reperfusion injury. The coronary ischemic diseased left ventricle (LV) undergoes gradual localized remodeling in response to chronic progressive ischemic disease whereas the LV with aortic valve stenosis (AVS) progresses into hypertrophy. Earlier clinical studies have indicated that hearts with hypertrophied LVs are at greater risk during cardiac operations than hearts of patients undergoing CABG surgery (2). This is further supported by work on animal hearts which have shown that, compared to normal myocardium, the hypertrophied LV is more vulnerable to reperfusion injury than normal hearts, whereas hearts with coronary artery disease were more resistant to the damaging effects of reperfusion injury (10, 11).

Following clinical evidence during the 1990s (12, 13), there was a shift from using cold crystalloid/blood cardioplegia to intermittent antegrade warm blood cardioplegia during CABG surgery. The superior cardioprotective efficacy with warm blood cardioplegia was associated with reduced changes in myocardial metabolites and free radical generation (14–17). In contrast, work involving AVS patients undergoing aortic valve replacement (AVR) suggested that cold blood is superior to warm blood cardioplegia (17, 18). Interestingly, and highlighting the importance of cold arrest, the use of different cold cardioplegic solutions with different composition in AVR patients produced similar protection (19). More recent studies confirmed this further and suggested that del Nido cardioplegia protocol is also an acceptable alternative for cold blood cardioplegia in patients undergoing AVR surgery (20, 21).

The cardioprotective efficacy of cardioplegic interventions can be influenced by the heterogeneity within the same heart especially between the two ventricular chambers which remodel differently in response to disease state in experimental models (22). Cardioplegic arrest in CABG and AVR patients includes both ventricular chambers where most cases will have one diseased chamber and one relatively normal one. Moreover, clinical studies have demonstrated that the metabolite levels in the LV with coronary disease differ from the LV of hearts with aortic stenosis, and the corresponding right ventricles (RVs) remodel significantly differently from LVs and from each other (9, 23). Compared to the LV of hearts with ischemic disease, hypertrophied hearts have higher levels of metabolites (e.g., ATP) which may have important implications for energy metabolism and protein turnover in the two pathologies (9). Therefore, the design of cardioplegic cardioprotective techniques must also consider the vulnerability of both the diseased (often the LV) and the relatively normal chamber (RV). In this context, intermittent cold blood cardioplegia during valve surgery is associated with more pronounced metabolic stress in the RV than the LV irrespective of the route of delivery (8).

Overall, studies investigating the effect of ischemic cardioplegic arrest during open heart surgery on cardiac metabolism including energy rich phosphates have largely focused on non-ventricular tissue or on diseased LV with few studies attempting to assess such changes in both LV and RVs. Moreover, whether such changes are altered in different pathologies or linked to reperfusion injury and inflammatory response is not presently known. In this study we report these changes for patients undergoing either CABG or AVR.

## Materials and methods

### Patients and tissue collection

The data presented in this work is a sub-study (post-hoc analysis) of a two-center randomized controlled trial investigating the effect of upper limb remote ischemic preconditioning (RIPC) in patients undergoing isolated coronary artery bypass grafting (CABG) or aortic valve replacement (AVR) on cardiac injury, metabolic stress and inflammatory responses (24, 25). The trial was conducted at the Hammersmith Hospital and the Bristol Royal Infirmary and was sponsored by the Imperial College of London. The study was registered (International Standard Randomized Controlled Trial Number 33084113).

Overall trial criteria for inclusion, exclusion and conduct have been previously published (24, 25). A group of patients who did not receive RIPC undergoing either CABG (*n* = 26) or AVR (*n* = 17) were randomly selected from the control group of the trial and used for this sub-study. Myocardial biopsies were obtained using a Trucut needle from the left and the right ventricles pre and 20 min after the end of cardioplegic arrest (post). Biopsies smaller than 0.5 mg (*n* = 6 biopsies) or larger than 10 mg (*n* = 1 biopsy) were excluded. Following this, patients with <3 biopsies each were excluded (*n* = 2 patients, one from each group). In total 157 biopsies were included in the analyses (94 from 25 CABG patients and 63 from 16 AVR patients).

### Anesthesia, surgery and cardioplegia management

Anesthetic management, cardiopulmonary bypass (CPB), cardioplegia, surgical techniques and any other aspect of pre- and post-operative management were in accordance with existing protocols at both centers. Surgery proceeded as per routine practice in each center. Cardioplegic ischemic arrest was induced using cold blood cardioplegia (4parts blood:1part cardioplegia ratio) given at a temperature of approximately 4°C as described in detail elsewhere (24, 25).

### Tissue processing

Collected frozen cardiac tissues were crushed using a pestle and mortar in liquid nitrogen. The resulting tissue powder was immediately moved into an LP3 tube containing 4.8% perchloric acid (PCA), cooled to 4°C. The tubes were weighed before and after adding the tissue powder to obtain wet tissue weight. The mixture was vortexed (Rotamixer 7871, Hook & Tucker Instruments) and centrifuged at 4°C, 4,000 g for 10 min using Allegra 21R centrifuge (Beckman Coulter, UK). The supernatant was neutralized by mixing 400 μl with equal volume of 0.44 M K_2_CO_3_. The mixture was then centrifuged again at 4°C, 4,000 g for 10 min. The supernatant was collected, aliquoted and stored at −20°C freezer for HPLC analysis.

### HPLC

High-performance liquid chromatography (HPLC) was used to separate and identify metabolites as reported previously (24, 26, 27). The instrumentation consisted of a solvent module pump, an autosampler, a separation column, and a detector all from Beckman Coulter Ltd, UK). The signals generated from HPLC were displayed as chromatograms using (OpenLab Software, Aligent, USA). Peaks of each metabolite in the sample chromatograms were identified using standards of known concentration. Data analysis was completed by measuring the area under the curve for each peak, and the values were corrected to account for the dilution factor and the weight of each biopsy in order to produce the concentration of each metabolite. Energy charge for adenylates has been used as a measure of metabolic flux rate (28) and is calculated as follows:


(1) $$\begin{array}{r} \begin{array}{c} Energy\;charge=\frac{ATP+\;0.5ADP}{ATP+ADP+AMP} \end{array} \end{array}$$

### Blood sample preparation and analysis

Markers of cardiac injury, inflammatory response and oxidative stress were measured in blood samples collected preoperatively (prior to CPB) and postoperatively at 1, 6, 12, 24, 48, and 72 h. Whole blood samples were coagulated with EDTA and centrifuged for 10 min at 1,000 × g within 30 min of collection. Plasma was removed and assayed immediately in accordance with kit manufacturer instructions or aliquoted for storage at −20°C until use. Details are shown elsewhere (24, 25). In brief, IL-6, IL-8, IL-10, TNFα and Myeloperoxidase (MPO) were measured using the Milliplex MAP Human High Sensitivity T Cell Magnetic Bead Panel (Millipore, Billerica, USA). Troponin I was measured as per standard clinical practice.

### Statistics

For the comparison of patient characteristics and metabolites measured by HPLC, the data were first tested for normality using the Shipiro-Wilk test. Normal data was analyzed with unpaired, 2-tailed *t*-tests whilst non-normal data was analyzed with Mann-Whitney U test. Levels of troponin, MPO and cytokines were analyzed by 2-way mixed model ANOVA with one repeated measures factor (time) and one between subjects factor (operation type). Area under the curve (AUC) was also calculated for each curve, tested for normality and compared between groups using 2-tailed *t*-tests or Mann-Whitney U tests as appropriate. A one-way ANOVA with post-hoc Dunnett's test where appropriate was also performed for each surgery type to compare which timepoints were significantly different from baseline. All statistics were performed in SPSS (Version 27, IBM Analytics, New York, USA) and significance was accepted when *p* < 0.05.

## Results

Patients' characteristics for both pathologies were similar in age and gender distribution (Table 1). Time spent on cardiopulmonary bypass (CPB) was slightly but not significantly more for the AVR group. However, and in general agreement with data from clinical practice, AVR patients had a significantly longer cross-clamp time compared to CABG group. Nearly half (*n* = 12) of the CABG patients were diabetic in contrast to only one in the AVR group. Table 2 shows the characteristics of CABG patients divided into diabetic and non-diabetic.

**Table 1:** Patient characteristics.

	**CABG (*****n** **=*** **25)**	**AVR (*****n** **=*** **16)**
Age (years)	62.0 ± 2.4	65.1 ± 3.4
Gender (M/F)	22/3	13/3
CPB time (minutes)	81.9 ± 3.9	91.1 ± 6.6
Cross clamp time (minutes)	38.6 ± 2.3	63.0 ± 4.9^*^
Diabetes (Y/N)	12/13	1/15
Hypertension (Y/N)	24/1	12/4

*Values are mean ± SEM*.

^*^represents a significant difference between groups (unpaired two-tailed student's t-test or Mann-Whitney U-test as appropriate). The age of one patient in the CABG group was not available.

**Table 2:** Characteristics of diabetic and non-diabetic CABG patients.

	**Non-diabetic (*****n** **=*** **13)**	**Diabetic (*****n** **=*** **12)**
Age (years)	58.8 ± 4.3	65.3 ± 2.0
Gender (M/F)	11/2	11/1
Diabetes: 0 I II III	13 0 0 0	0 0 9 (NIDDM) 3 (IDDM)
Hypertension (Y/N)	12/1	12/0
CPB time (minutes)	86.4 ± 5.6	77.0 ± 5.1
Cross clamp time (minutes)	40.9 ± 3.3	36.1 ± 3.1

*Values are mean ± SEM. There were no significant differences between groups (unpaired two-tailed student's t-test or Mann-Whitney U-test as appropriate). NIDDM, non-insulin-dependent diabetes mellitus; IDDM, insulin-dependent diabetes mellitus. The age of one patient in the non-diabetic group was not available*.

### Changes in cardiac metabolites of LVs and RVs in CABG and AVR patients undergoing surgery

Table 3 shows the cardiac concentrations of metabolites in left and right ventricular chambers of both CABG and AVR patients at basal level and 20 min during reperfusion after ischemic cardioplegic arrest. The concentrations of metabolites tended to be lower at basal levels in both LV and RV of CABG patients compared to the corresponding ventricle of AVR patients; ATP, GTP and NAD were significantly lower in CABG LV compared to LV of AVR patients whilst ATP, ADP, AMP, GTP, GDP, NAD, and glutamate were all significantly lower in the RV of CABG patients compared to RV in AVR patients (Table 3).

**Table 3:** Concentration of metabolites before and after surgery in LV and RV of CABG and AVR patients.

	**CABG (*****n** **=*** **25)**						**AVR (*****n** **=*** **16)**					
**Metabolite (nmol/ mg)**	**LV**			**RV**			**LV**			**RV**		
	**Pre**	**Post**	***p***	**Pre**	**Post**	***p***	**Pre**	**Post**	***p***	**Pre**	**Post**	***p***
AMP	1.08 ± 0.17	0.73 ± 0.10	0.054	0.56 ± 0.05	0.61 ± 0.10	0.704	1.12 ± 0.17	0.79 ± 0.14	0.080	0.96 ± 0.13^¥^	0.83 ± 0.15	0.299
ADP	1.95 ± 0.23	1.30 ± 0.19^*^	0.005	1.16 ± 0.11	1.06 ± 0.15	0.368	2.37 ± 0.22	1.66 ± 0.19^*^	0.021	2.00 ± 0.18^¥^	1.61 ± 0.21	0.172
ATP	1.94 ± 0.25	1.02 ± 0.18^*^	0.005	1.32 ± 0.21	0.99 ± 0.16	0.222	3.05 ± 0.29^≠^	2.17 ± 0.35	0.062	2.88 ± 0.29^¥^	1.74 ± 0.25^*^	0.006
GMP	0.05 ± 0.01	0.04 ± 0.02	0.467	0.03 ± 0.01	0.04 ± 0.01	0.710	0.05 ± 0.02	0.02 ± 0.01	0.682	0.02 ± 0.01	0.03 ± 0.02	0.922
GDP	0.11 ± 0.02	0.07 ± 0.01^*^	0.020	0.06 ± 0.01	0.06 ± 0.01	0.850	0.12 ± 0.01	0.08 ± 0.01^*^	0.041	0.09 ± 0.01^¥^	0.07 ± 0.01	0.231
GTP	0.12 ± 0.02	0.07 ± 0.01^*^	0.013	0.07 ± 0.01	0.06 ± 0.01	0.476	0.24 ± 0.02^≠^	0.17 ± 0.03	0.055	0.16 ± 0.02^¥^	0.11 ± 0.02	0.101
Adenosine	0.41 ± 0.08	0.34 ± 0.05	0.874	0.31 ± 0.04	0.34 ± 0.06	0.984	0.25 ± 0.03	0.24 ± 0.03	0.515	0.30 ± 0.04	0.33 ± 0.06	0.830
Hypoxanthine	0.19 ± 0.05	0.21 ± 0.06	0.496	0.18 ± 0.05	0.17 ± 0.03	0.657	0.11 ± 0.05	0.07 ± 0.02	0.216	0.09 ± 0.04	0.19 ± 0.12	0.775
Alanine	3.15 ± 0.31	3.44 ± 0.46	0.897	2.81 ± 0.33	3.20 ± 0.45	0.447	3.02 ± 0.45	3.16 ± 0.38	0.515	3.20 ± 0.55	4.54 ± 0.97	0.247
Inosine	0.48 ± 0.13	0.52 ± 0.14	0.401	0.39 ± 0.07	0.31 ± 0.04	0.610	0.41 ± 0.10	0.34 ± 0.05	0.985	0.40 ±0.07	0.56 ± 0.22	0.545
βNAD	0.51 ± 0.08	0.31 ± 0.04^*^	0.007	0.30 ± 0.03	0.24 ± 0.03	0.208	0.60 ± 0.06^≠^	0.43 ± 0.05^*^	0.029	0.54 ± 0.05^¥^	0.43 ± 0.08	0.054
Glutamate	10.36 ± 1.23	5.47 ± 0.81^*^	6.2 × 10^−5^	6.28 ± 0.68	4.31 ± 0.44^*^	0.012	12.29 ± 0.99	7.52 ± 1.14^*^	0.004	9.39 ± 0.92^¥^	6.09 ± 0.86^*^	0.006
IMP	0.86 ± 0.28	0.63 ± 0.23	0.525	0.63 ± 0.16	0.55 ± 0.15	0.570	0.56 ± 0.36	0.19 ± 0.08	1.000	0.38 ± 0.20	0.44 ± 0.31	0.495

*Values are mean ± SEM. Statistical significance was assessed using unpaired two-tailed student's t-test or Mann-Whitney U test as appropriate. Underlined p-values indicate borderline significance*.

* *significant difference vs. corresponding Pre sample*.

≠ *significant difference vs. Pre values in CABG LV*.

¥ *significant difference vs. Pre values in CABG RV*.

*Bold p-values are significant (p < 0.05)*.

**Figure 1:**
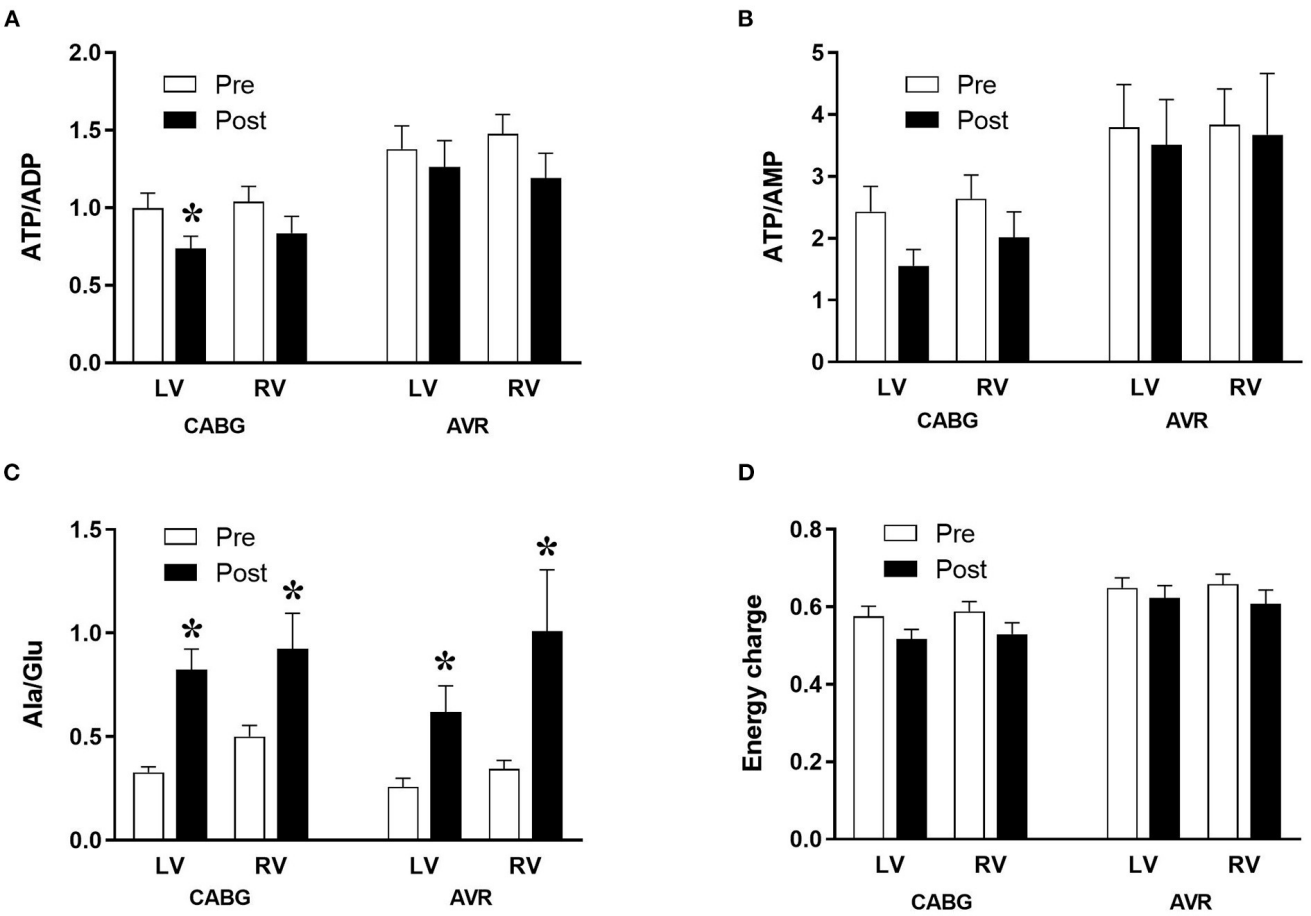
Metabolic ischemic stress in CABG and AVR patients. Values are mean ± SEM for ATP/ADP **(A)**, ATP/AMP **(B)**, Ala/Glu **(C)**, and Energy charge **(D)** in left (LV) and right (RV) ventricles of patients pre- (white) and post- (black) cardioplegic arrest in CABG or AVR surgery. ^*^Represents a significant difference vs. corresponding pre samples (unpaired two-tailed student's *t*-test or Mann-Whitney *U*-test as appropriate).

The changes in metabolites due to ischemia and reperfusion are more extensive and evident in the LV of CABG patients compared to the corresponding RV and to both LV and RV of AVR patients. Unlike the RV of CABG patients, the LV showed a significant fall in several key metabolites including ATP, ADP, GTP, GDP and βNAD. There were fewer changes in both ventricles of the AVR group where the LV showed a fall in ADP, GDP, and βNAD whilst the RV showed a significant fall in ATP. The amino acid glutamate levels decreased in all ventricles of both pathologies. Figure 1 shows the computed values that are used to estimate ischemic stress (29). It is evident that all four chambers in both pathologies had a significant metabolic stress as assessed using the alanine/glutamate ratio (30). The energy charge after surgery was not significantly different from basal levels although there were strong trends in the CABG patients. Energy charge correlates well with the activity of enzymes involved in utilization of ATP and has previously been used as a measure of metabolic flux rate (28). However, only the LV of CABG patients had a significant fall in the phosphorylation potential (ATP/ADP) indicating reduced ability to make ATP (29).

### Diabetes and changes in cardiac metabolites in CABG patients

**Figure 2:**
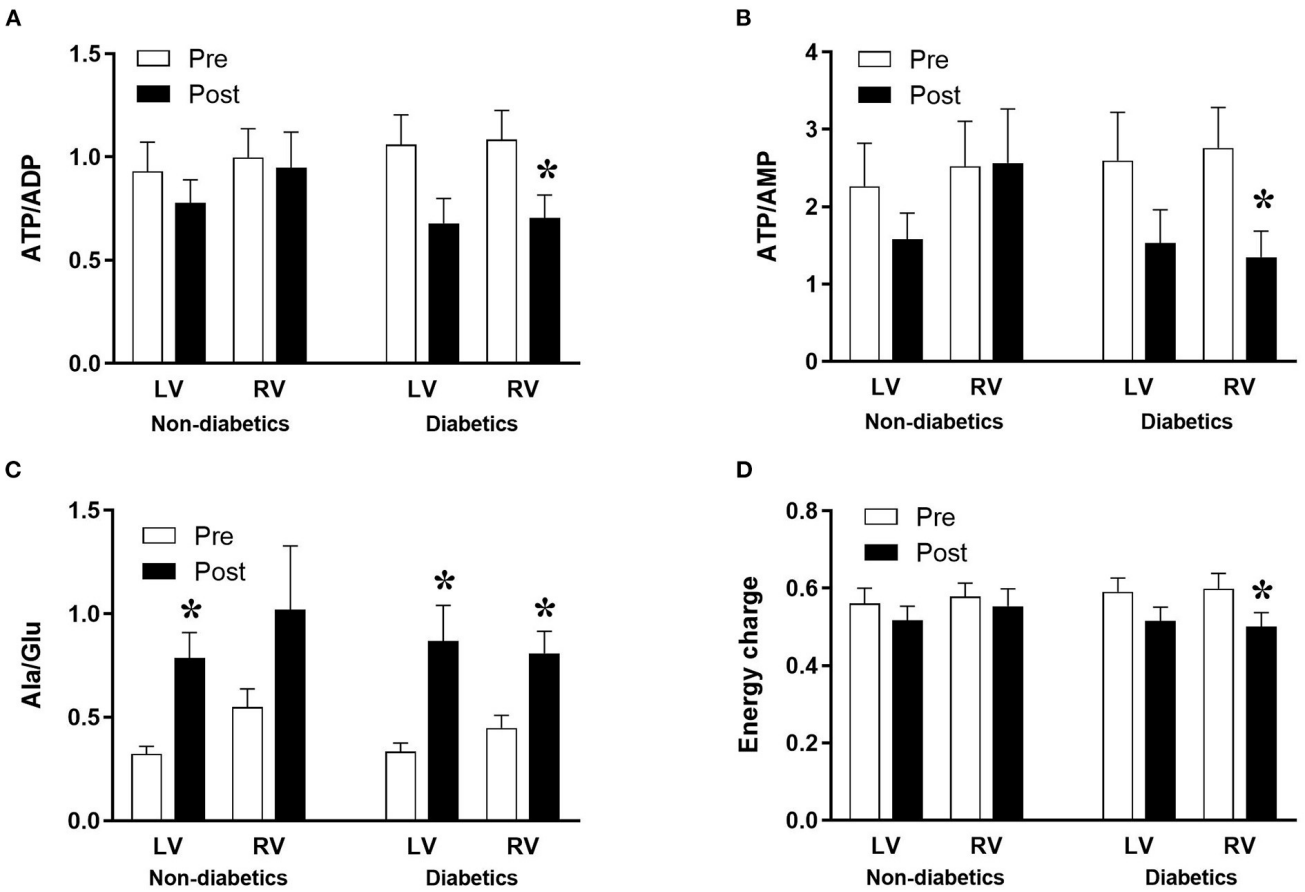
Diabetes and metabolic ischemic stress during surgery in CABG patients. Values are mean ± SEM for ATP/ADP **(A)**, ATP/AMP **(B)**, Ala/Glu **(C)**, and Energy charge **(D)** in left (LV) and right (RV) ventricles of patients Pre- (white) and Post- (black) CABG surgery who are non-diabetic or diabetic. *Represents a significant difference vs. corresponding Pre samples (unpaired two-tailed student's *t*-test or Mann-Whitney *U*-test as appropriate).

The data above showed marked and significant changes in the LV of CABG patients compared to all other chambers in both groups. In addition to the coronary disease, a significant number of CABG patients were also diabetic thus adding an important co-morbidity that can potentially alter the levels of metabolites. Table 4 shows the metabolic changes in CABG group when divided into diabetic (*n* = 12) and non-diabetic patients (*n* = 13). The LV of both diabetic and non-diabetic patients showed significant fall (or strong trend) in several metabolites including ATP, ADP and glutamate. There was no change in the RVs of diabetic and non-diabetic patients apart from glutamate, which fell significantly in the RV of diabetic patients. All chambers except the RV of non-diabetics showed a significant increase in the alanine/glutamate ratio, whilst a decrease in other markers of ischemic metabolic stress [ATP/ADP, ATP/AMP and energy charge (suggesting reduced metabolic flux)] was seen during early reperfusion only in the RV of the diabetic patients (Figure 2).

**Table 4:** Concentration of metabolites before and after surgery in LV and RV of diabetic and non-diabetic CABG patients.

**Metabolite (nmol/ mg)**	**Diabetic (*****n =*** **12)**						**Non-Diabetic (*****n =*** **13)**					
	**LV**_**pre**_	**LV**_**post**_	***p***	**RV**_**pre**_	**RV**_**post**_	***p***	**LV**_**pre**_	**LV**_**post**_	***p***	**RV**_**pre**_	**RV**_**post**_	***p***
AMP	1.00 ± 0.12	0.88 ± 0.19	0.426	0.63 ± 0.09	0.75 ± 0.21	0.786	1.16 ± 0.34	0.61 ± 0.09	0.13	0.50 ± 0.04	0.50 ± 0.07	0.95
ADP	1.94 ± 0.16	1.60 ± 0.34	0.099	1.31 ± 0.20	1.21 ± 0.29	0.765	1.97 ± 0.45	1.06 ± 0.19	0.03	1.02 ± 0.10	0.93 ± 0.11	0.55
ATP	2.14 ± 0.37	1.08 ± 0.23	0.028	1.52 ± 0.35	0.96 ± 0.25	0.219	1.74 ± 0.35	0.97 ± 0.27	0.07	1.14 ± 0.24	1.01 ± 0.22	0.69
GMP	0.04 ± 0.01	0.06 ± 0.04	0.918	0.03 ± 0.01	0.04 ± 0.01	0.880	0.06 ± 0.02	0.02 ± 0.005	0.30	0.03 ± 0.01	0.03 ± 0.01	0.77
GDP	0.10 ± 0.02	0.09 ± 0.03	0.349	0.06 ± 0.01	0.06 ± 0.02	0.945	0.13 ± 0.04	0.05 ± 0.01	0.03	0.06 ± 0.01	0.06 ± 0.01	0.84
GTP	0.12 ± 0.02	0.08 ± 0.02	0.081	0.09 ± 0.02	0.05 ± 0.02	0.187	0.13 ± 0.04	0.06 ± 0.01	0.07	0.06 ± 0.01	0.07 ± 0.01	0.73
Adenosine	0.43 ± 0.12	0.40 ± 0.08	0.973	0.31 ± 0.05	0.46 ± 0.13	0.525	0.39 ± 0.10	0.30 ± 0.05	0.77	0.31 ± 0.07	0.24 ± 0.03	0.88
Hypoxanthine	0.10 ± 0.05	0.29 ± 0.13	0.099	0.19 ± 0.10	0.14 ± 0.04	0.260	0.28 ± 0.09	0.15 ± 0.04	0.45	0.18 ± 0.04	0.20 ± 0.05	0.77
Alanine	3.02 ± 0.46	4.30 ± 0.96	0.393	2.91 ± 0.51	3.28 ± 0.92	1.000	3.25 ± 0.45	2.77 ± 0.28	0.35	2.72 ± 0.44	3.13 ± 0.34	0.46
Inosine	0.27 ± 0.11	0.77 ± 0.31	0.043	0.41 ± 0.13	0.32 ± 0.05	0.487	0.69 ± 0.23	0.33 ± 0.04	0.16	0.37 ± 0.05	0.30 ± 0.06	0.31
βNAD	0.48 ± 0.05	0.36 ± 0.07	0.152	0.31 ± 0.04	0.26 ± 0.06	0.477	0.55 ± 0.15	0.27 ± 0.05	0.04	0.28 ± 0.03	0.23 ± 0.03	0.28
Glutamate	9.42 ± 1.15	6.53 ± 1.51	0.043	7.24 ± 1.25	4.07 ± 0.75^*^	0.037	11.22 ± 2.13	4.65 ± 0.81	0.001	5.40 ± 0.56	4.51 ± 0.53	0.26
IMP	0.52 ± 0.25	0.79 ± 0.48	0.809	0.46 ± 0.14	0.26 ± 0.09	0.413	1.20 ± 0.50	0.51 ± 0.18	0.25	0.78 ± 0.28	0.82 ± 0.25	0.81

*Values are mean ± SEM*.

* *Represents significant difference vs. corresponding Pre sample, unpaired two-tailed student's t-test or Mann-Whitney U test as appropriate. Underlined p-values indicate borderline significance. Bold p-values are significant (p < 0.05)*.

### Changes in markers of cardiac injury and oxidative stress in CABG and AVR patients undergoing surgery

**Figure 3:**
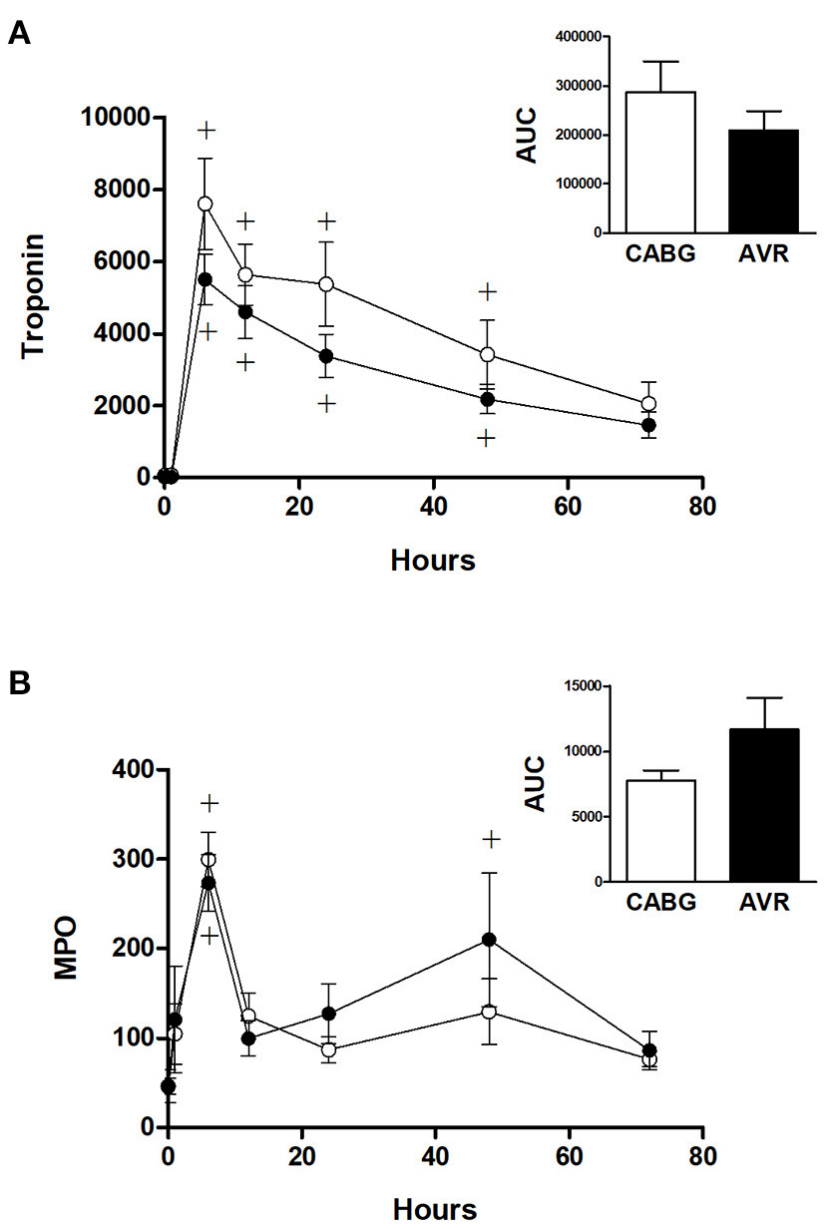
Cardiac injury and systemic oxidative stress in CABG and AVR patients. Pre- and post-operative levels of **(A)** troponin I (ng/L) and **(B)** MPO (ng/ml) in patients undergoing CABG (white) or AVR (black) surgery. Values are mean ± SEM for CABG or AVR. Two-way mixed model ANOVA for **(A)** revealed a significant interaction between time and operation type but no significance differences *from post-hoc* testing and for **(B)** revealed no significant differences. One-way ANOVAs with post-hoc Dunnett's test where appropriate were also performed individually for each surgery type; + represents a significant difference compared to basal levels within the same group. No statistical significance was found for the AUC (area under the curve) analyses (unpaired two-tailed student's *t*-test or Mann-Whitney *U*-test as appropriate).

Figure 3A shows the time dependent change in blood cardiac troponin I (cTnI) during open heart surgery for both CABG and AVR patients. The plasma cTnI levels increased to reach a maximum at 6 h post operatively followed by a gradual decrease over several days postoperatively. The increase in cTnI levels was significantly different at time points up to 48 h compared to basal levels which was the same for both CABG and AVR groups. Although the CABG group tended to have higher values than AVR, these were not statistically significant even when presented as total release (area under the curve; Figure 3 inset).

Figure 3B shows the time dependent change in blood myeloperoxidase (MPO), a marker of oxidative stress. In both CABG and AVR groups, plasma MPO levels increased significantly at 6 h postoperatively followed by a decline where there was no difference at 12 h compared to preoperative levels. The increase in MPO levels was similar for both groups although when presented as total release (area under the curve) there was a trend for CABG to have lower levels than AVR (Figure 3B inset).

### Effect of diabetes on markers of cardiac injury and oxidative stress in CABG patients

**Figure 4:**
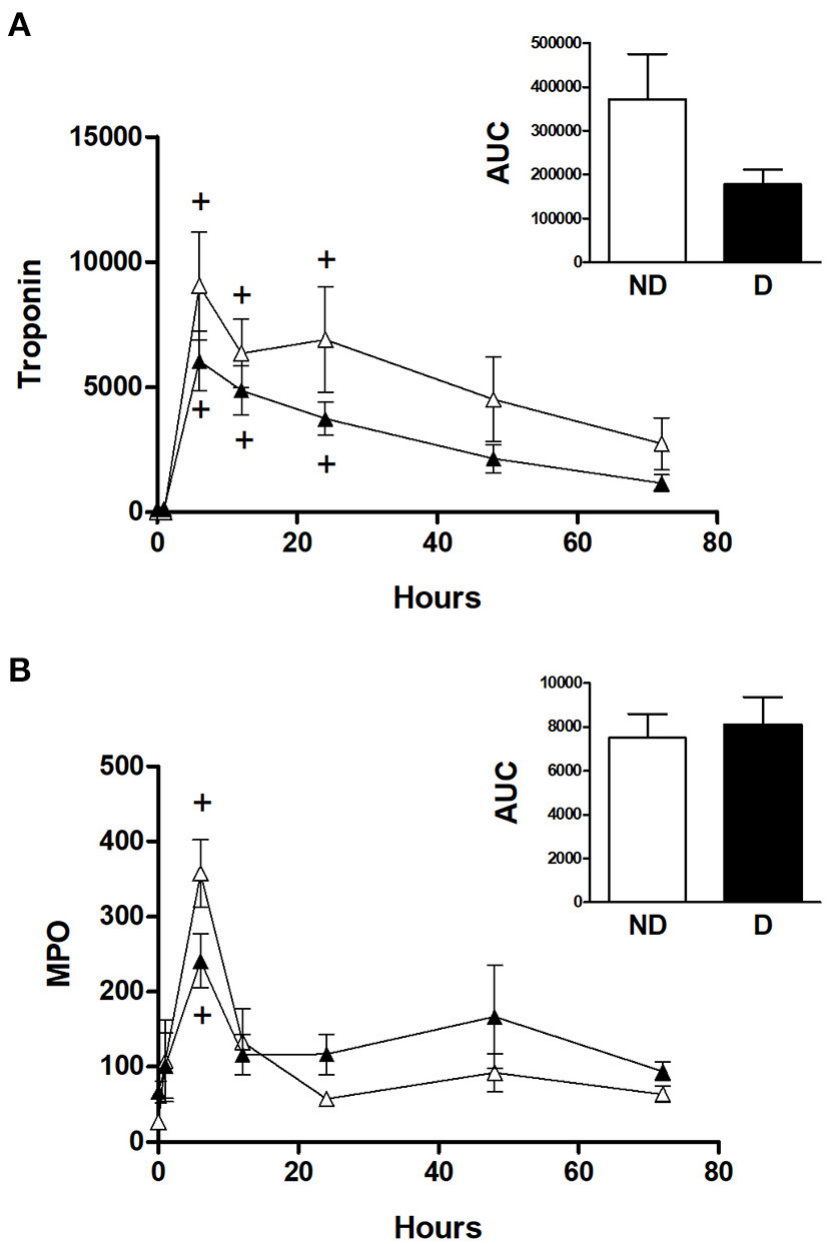
Cardiac injury and oxidative stress in diabetic and non-diabetic CABG patients. Values are mean ± SEM for 'levels of **(A)** troponin I (ng/L) and **(B)** MPO (ng/ml) in patients undergoing CABG surgery who are non-diabetic (ND, white) or diabetic (D, black). No significant differences were revealed by two-way mixed model ANOVA. One-way ANOVAs *with post-hoc* Dunnett's test where appropriate were also performed individually for each surgery type; + represents a significant difference compared to basal levels. No statistical significance was found for the AUC (area under the curve) analyses (unpaired two-tailed student's *t*-test or Mann-Whitney *U*-test as appropriate).

Figure 4 shows the cTnI and MPO data for diabetic and non-diabetic patients within the CABG group. Analysis for the effect of diabetes within the CABG group shows that diabetic patients tended to have relatively less release of cTnI; there was a trend for a reduced AUC in the diabetic patients but this was not statistically significant (*p* = 0.522) due to large variability in the non-diabetic group (Figure 4A). The release of MPO was not affected by diabetes (Figure 4B).

### Changes in inflammatory markers in CABG and AVR patients undergoing surgery

**Figure 5:**
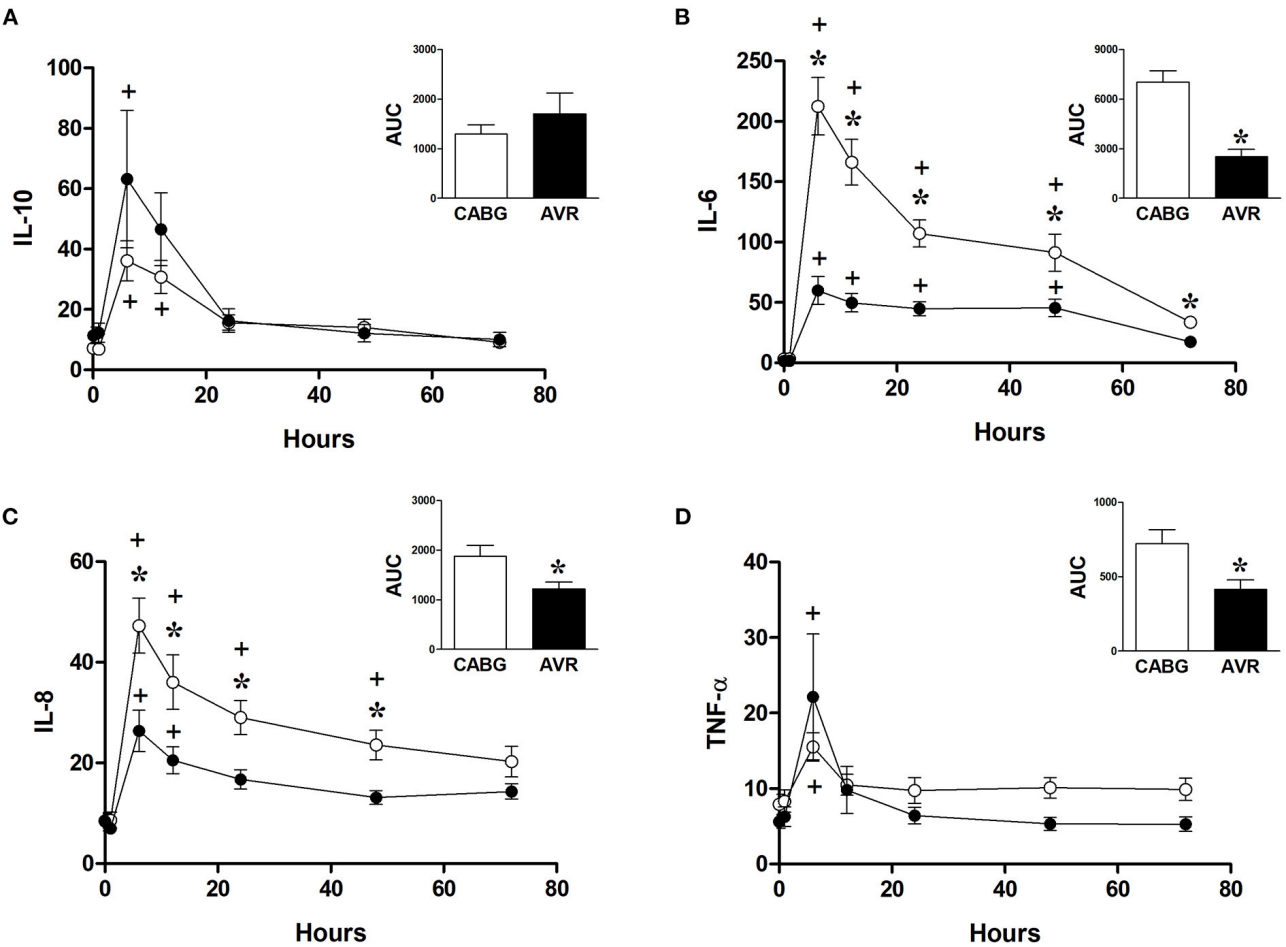
Postoperative changes in inflammatory markers in CABG and AVR patients. Values (pg/ml) are mean ± SEM for levels of IL-10 **(A)**, IL-6 **(B)**, IL-8 **(C)**, and TNF-α **(D)**, in patients undergoing CABG (white) or AVR (black) surgery. Analysis by two-way mixed model ANOVA did not reveal any significant differences for **(A)** or **(D)**, whilst in **(B)**, and **(C)** a significant interaction was found between time and operation type; * represents a significant effect of operation type (CABG vs. AVR) at individual timepoints. One-way ANOVAs *with post-hoc* Dunnett's test where appropriate were also performed individually for each surgery type; + represents a significant difference compared to basal levels. AUC (area under the curve) was analyzed using unpaired two-tailed student's *t*-test or Mann-Whitney *U*-test as appropriate; *Represents a significant difference between groups.

Figure 5 shows the changes in the systemic levels of inflammatory cytokines in the two groups of patients. IL-6 and IL-8 in both CABG and AVR groups followed similar patterns where a rise in their levels reached a peak at 6 h postoperatively before starting to decline over the remaining postoperative period. For both groups of patients, the increase in IL-6 was significantly different from basal levels at different time points up to 48 h postoperatively (Figure 5B). Furthermore, the levels of IL-6 were significantly higher in CABG patients compared to AVR patients at all timepoints between 6–72 h postoperatively. When data were expressed as total release the amount was approximately three-fold higher in the CABG group compared to the AVR group (Figure 5B inset). The trends and changes in IL-6 were also seen for IL-8 which was significantly higher in CABG at 6, 12, 24, and 48 h postoperatively (Figure 5C), and the total IL-8 release was significantly difference (Figure 5C inset). TNF-α levels also peaked at 6 h (Figure 5D), and the total release was lower in AVR patients (Figure 5D inset). IL-10 values increased significantly at 6 and 12 h in CABG patients but only at 6 hour in AVR patients (Figure 5A). Although the initial increase in IL-10 levels in AVR appeared more than CABG, the total release was not significantly different (Figure 5A inset).

### The effect of diabetes on changes in inflammatory markers in CABG patients

**Figure 6:**
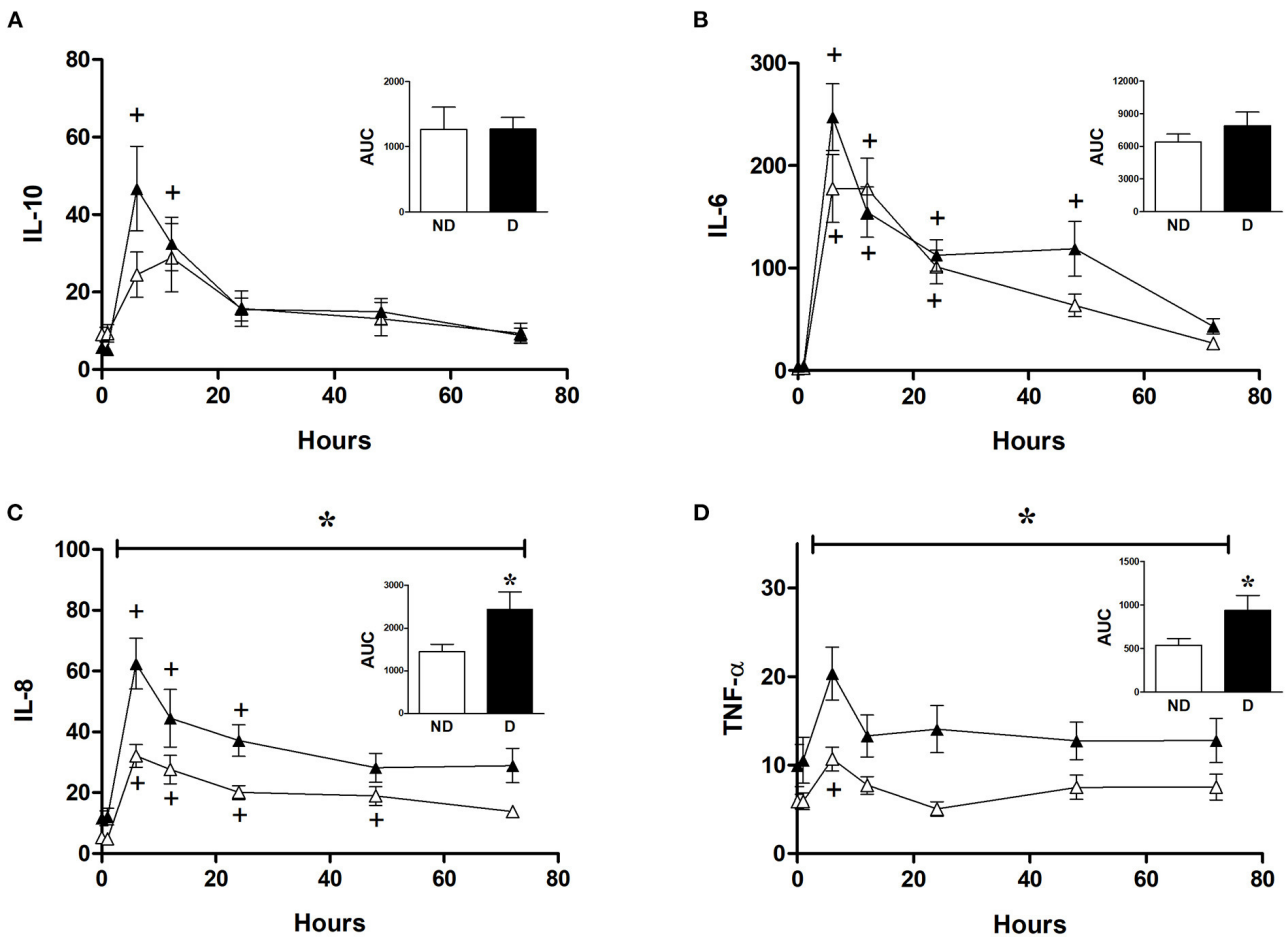
Effect of diabetes on postoperative changes in inflammatory markers. Values (pg/ml) are mean ± SEM for levels of IL-10 **(A)**, IL-6 **(B)**, IL-8 **(C)**, and TNF-α **(D)**, in patients undergoing CABG surgery who are non-diabetic (ND, white, *n* = 12) or diabetic (D, black, *n* = 12). Analysis by two-way mixed model ANOVA did not reveal any significant differences for **(A)** or **(B)**, whilst the horizontal bracket in **(C)** and **(D)** represents a significant “between subjects effect” over all timepoints. One-way ANOVAs *with post-hoc* Dunnett's test were appropriate were also performed individually for non-diabetic and diabetic patients; + represents a significant difference compared to basal levels. AUC (area under the curve) was analyzed using unpaired two-tailed student's *t*-test or Mann-Whitney *U*-test as appropriate; *Represents a significant difference between groups.

Analysis of the role of diabetes in CABG patients shows that diabetic patients had a significantly higher release of IL-8 and TNFα compared to non-diabetic patients (Figures 6C,D). However, diabetes did not significantly alter the release of IL-6 and IL-10 in CABG patients (Figures 6A,B).

## Discussion

This study reports novel data showing the metabolic/energetic changes in both diseased LV and relatively normal RV of two major pathologies for patients undergoing cardiac surgery using cold blood cardioplegic arrest. The duration of ischemic arrest for CABG surgery was significantly shorter than AVR group (Table 1) which would suggest reduced metabolic changes and reperfusion injury. However, the data clearly suggest otherwise and confirms earlier suggestions that different disease states respond differently to ischemia/reperfusion (please see introduction). Therefore, the discussion will address the changes in cardiac metabolites in both ventricular chambers and the associated systemic markers of injury/inflammation/stress in the context of differences in pathology.

### Reperfusion of diseased LVs is associated with sustained fall in their metabolites

Consistent with earlier studies (8, 9) we find similar basal levels of ATP in LV and RV of AVR patients and that these levels are higher than those for LV of CABG patients (Table 3). Additionally, we now report that ATP levels (and most of the measured metabolites) in the RV of AVR patients are also higher than RV of CABG patients (Table 3). These findings confirm earlier suggestions that the LV with coronary disease has evidence of greater metabolic/ischemic stress compared to LV with aortic disease (9). Interestingly, we find that this difference is also seen in the relatively normal RV indicating metabolic remodeling in one ventricle alters remodeling in the adjacent one.

Earlier work in 1985 using rodent hearts has demonstrated that the ischemia-induced fall in ATP and other metabolites is not reversed early after reperfusion despite resumption of cardiac function (31). Furthermore, poor functional recovery during reperfusion is associated with depressed ATP/ADP and ATP/AMP ratios (32). Similar observations have been made in studies involving hearts of patients undergoing CABG or AVR surgery using cold ischemic cardioplegic arrest which have also reported depressed levels of ventricular metabolites and increased markers of metabolic stress (17, 33, 34). In the current study, the changes in metabolites due to ischemia and reperfusion are more extensive and evident in the LV of CABG patients compared to the RV or to both ventricles of AVR patients—for example we found that the phosphorylation potential (as measured by the ATP/ADP ratio) was significantly decreased only in the LV of CABG patients. This is consistent with earlier work on CABG patients where cold blood cardioplegic arrest was associated with a sustained fall in ATP and glutamate after reperfusion (34). Our ATP and ADP data for LV of AVR patients (Table 3) are similar to earlier reports using cold blood cardioplegic arrest (18). The finding that the fall in ATP is more evident in the RV compared to LV of AVR patients is also similar to earlier studies (8). The significant decrease in the amino acid glutamate in left and right ventricles of both pathologies and the subsequent increase in the alanine/glutamate ratio in all four ventricles confirms significant metabolic stress during cardioplegic arrest and early reperfusion (18, 30).

In summary, a sustained fall in cardiac metabolites seen during early reperfusion occurs mostly in the diseased LVs of CABG and AVR hearts with limited changes in the RVs. These observations suggest that the relatively normal RVs are either resistant to change during ischemia (not supported by earlier studies) or that these ventricles are able to quickly reverse ischemic-induced fall in metabolites. Despite the significantly shorter period of ischemic arrest, the marked metabolic changes in the LV of CABG patients suggest that coronary diseased hearts have a greater susceptibility to injury. However, evidence for this must come from markers of cardiac injury.

### CABG patients undergoing open heart surgery are more vulnerable to injury and inflammatory response compared to AVR patients

Cardiac injury (cTnI release) and systemic oxidative stress (MPO level) were similar for both CABG and AVR groups. However, the shorter ischemic arrest for CABG patients would suggest that vulnerability to reperfusion injury is relatively higher for CABG patients. This is consistent with the extent of the metabolic changes (see above) and suggests that coronary diseased hearts are more vulnerable to cold ischemic cardioplegic arrest and reperfusion than the AVR heart. This finding is different from our earlier experimental studies where we showed that coronary diseased mouse hearts were more resistant to ischemia/reperfusion injury whereas rat hearts with hypertrophied LV were more vulnerable to ischemia/reperfusion (10, 11). However, unlike the present clinical study, the experimental studies were carried out using normothermic arrest. We and others have shown that hearts of CABG patients sustain more injury when using cold cardioplegic arrest compared to intermittent warm cardioplegia whereas AVR hearts were better protected using cold cardioplegic arrest (12–18). Therefore, a comparative study between the two pathologies using warm blood cardioplegia will confirm this interpretation.

Unlike reperfusion injury, the inflammatory response was very marked and higher for CABG patients compared to AVR patients even when the cross-clamp is significantly shorter (Table 1). It is widely accepted that the main trigger of inflammation is the CPB duration (35, 36) which also tended to be shorter for CABG (Table 1). In addition to CPB, the cardiac ischemic arrest and reperfusion are also associated with inflammation which would contribute to the overall inflammatory response. In fact, cardiomyocytes are known to produce IL-6 during ischemia with or without CPB (37, 38). The importance of the pathology has been addressed where it has been reported that, in the absence of CPB, cardiac ischemia of hearts with coronary disease triggers increase in plasma IL-6 levels and the degree of IL-6 release is linked to the intensity of the coronary disease (39). It is important to note that basal levels of inflammatory markers were similar for both CABG and AVR patients. Therefore, the differences observed in this study are likely to be associated with the response of the two pathologies to surgery.

Coronary disease is an inflammatory disease and atherosclerosis can be diffuse and present throughout the body. More importantly, and unlike AVR patients, almost 50% of CABG patients were diabetic. Diabetes is a major co-morbidity and is often seen in CABG patients which could alter the susceptibility to ischemic arrest and reperfusion and the subsequent inflammatory response.

### Diabetes is implicated in the inflammatory response in CABG patients

There was no difference in cross clamp or CPB time between diabetic and non-diabetic CABG patients (Table 2). The basal levels of metabolites in LV and RV of both diabetic and non-diabetic patients are similar (Table 4). Additionally, the basal levels of markers of ischemic/metabolic stress in LV or RV of diabetic patients were similar to those of non-diabetic patients (Figure 2). Upon reperfusion, the RV of diabetic patients had significant and marked changes in all markers of ischemic/metabolic stress (Figure 2), whilst most stress markers in the RV of non-diabetics and the LV of both groups were not significantly different to basal levels (Figure 2). Similarly, markers of cardiac injury and oxidative stress were similar for both diabetic and non-diabetics (Figure 4), indicating vulnerability to ischemia reperfusion is similar for hearts from both diabetic and non-diabetic CABG patients. However, the main difference between diabetic and non-diabetic patients was the inflammatory response. Two of the inflammatory markers IL-8 and TNFα were significantly and markedly higher in diabetic patients compared to non-diabetics (Figure 6). This finding may account for some of the differences between CABG and AVR patients and suggests that diabetes is associated with an augmentation in the inflammatory response generated by cardioplegic arrest and reperfusion in addition to the CPB. It is difficult from this study to suggest whether the difference is due to reperfusion injury or CPB or both. A recent study using a rat model investigated the effect of extracorporeal membrane oxygenation and demonstrated that diabetes enhances the proinflammatory cytokine release (40). In fact it has been suggested that avoiding the use of CPB for diabetic patients with coronary disease will attenuate the incidence of organ complications (41).

### Limitations

The data presented in this work is a sub-study (post-hoc analysis) of a two-center randomized controlled trial. Therefore, there were restrictions in the design particularly in relation to patients and tissues collected. It would have been informative to have access to more tissue to investigate markers of inflammation, oxidative stress or apoptosis in the cardiac biopsies. With a larger number of patients, it would be possible to study the effects of gender, hypertension, diabetes, and other factors in both groups of patients. This should be the aim of a future study.

## Summary and conclusions

Exposure of the coronary diseased LV to chronic ischemic disease causes metabolic remodeling which differs from metabolic remodeling in the hypertrophied LV of aortic stenosis patients. Despite data indicating that the coronary diseased heart is preconditioned and more resistant to ischemic reperfusion injury, this work shows clearly that using cold blood cardioplegia confers less cardioprotection in patients with coronary disease compared to the hypertrophic hearts which are widely thought to be more vulnerable. This is in agreement with extensive studies which support the view that warm blood cardioplegia is superior to cold blood cardioplegia in hearts with coronary disease but not in hearts undergoing surgery for aortic valve disease. The inflammatory response in CABG patients is likely to be at least partly due to a high incidence of diabetes in CABG patients.

## Data availability statement

The raw data supporting the conclusions of this article will be made available by the authors, without undue reservation.

## Ethics statement

The studies involving human participants were reviewed and approved by London-Harrow Research Ethics Committee (REC Number 12/LO/1361). The patients/participants provided their written informed consent to participate in this study.

## Author contributions

Conceptualization of the study was achieved by M-SS, GA, and BR. Funding acquisition was acquired by CE, BR, GA, PP, and M-SS. The research methodology was carried out by MM, FF, BR, GA, and M-SS. Formal analysis was conducted by KS and SA-G. Writing of the original draft was performed by KS and M-SS. Review and editing was conducted by BR, GA, and M-SS. All authors read and approved the manuscript.

## Funding

This study was funded/supported by the British Heart Foundation (RG/15/5/31446) and the NIHR Biomedical Research Centre at University Hospitals Bristol NHS Foundation Trust and the University of Bristol (UK).

## Conflict of interest

The authors declare that the research was conducted in the absence of any commercial or financial relationships that could be construed as a potential conflict of interest.

## Publisher's note

All claims expressed in this article are solely those of the authors and do not necessarily represent those of their affiliated organizations, or those of the publisher, the editors and the reviewers. Any product that may be evaluated in this article, or claim that may be made by its manufacturer, is not guaranteed or endorsed by the publisher.

## Abbreviations

AUC: Area under the curve
AVR: Aortic valve replacement
CABG: Coronary artery bypass graft
CPB: Cardiopulmonary bypass
IDDM: Insulin-dependent diabetes mellitus
LV: Left ventricle
NIDDM: Non-insulin-dependent diabetes mellitus
RIPC: Remote ischaemic preconditioning
RV: Right ventricle.
